# Supplementary material for: Genome-wide identification, evolution, and expression analysis of the NPR1-like gene family in pears
Source: PeerJ. 2021 Dec 15;9:e12617. doi: 10.7717/peerj.12617 (PMC8684321; doi:10.7717/peerj.12617)
Supplement: Supplemental Information 1 [file peerj-09-12617-s001.docx]

**Table S 1. Information about the NPR1-like genes in various plants**

| **Class** | Species | Gene name | Accession number/Locus name | DOI |
| --- | --- | --- | --- | --- |
| **Monocots** | Oryza sativa | OsNPR1/NH1 | DQ450948 | https://doi.org/10.1111/j.1467-7652.2007.00243.x |
|  |  | OsNPR2/NH2 | DQ450949 |  |
|  |  | OsNPR3/NH3 | DQ450952 |  |
|  |  | OsNPR5/NH5 | DQ450956 |  |
|  | Musa acuminata | MNPR1A | DQ925843 | https://doi.org/10.1016/j.plaphy.2008.06.007 |
|  |  | MNPR1B | EF137717 |  |
|  | Gladiolus hybridus | GhNPR1 | AIM54370.1 | https://doi.org/10.1007/s00299-015-1765-1 |
|  | Lilium | LhSorNPR1 | APG55777.1 | https://doi.org/10.1007/s12298-017-0466-3 |
|  | Triticum aestivum L. | TaNPR1 | TraesCS3A02G105400.1 | https:// doi.org/10.3390/ijms20235974 |
|  |  | TaNPR2 | TraesCS4A02G470500.1_ |  |
|  |  | TaNPR3 | TraesCS3A02G298800.1 |  |
|  |  | TaNPR4 | TraesCS4A02G294400.1 |  |
|  |  | TaNPR5 | TraesCS3A02G489000.1 |  |
|  |  | TaNPR6 | TraesCS5A02G134700.2 |  |
| **Dicots** | Arabidopsis thaliana | AtNPR1 | At1g64280 | https://doi.org/10.1111/j.1365-313X.2006.02903.x |
|  |  | AtNPR2 | At4g26120 |  |
|  |  | AtNPR3 | At5g45110 |  |
|  |  | AtNPR4 | At4g19660 |  |
|  |  | AtNPR5/BOP2 | At2g41370 |  |
|  |  | AtNPR6/BOP1 | At3g57130 |  |
|  | Nicotiana tabacum | NtNPR1 | AAM62410 | <https://doi.org/10.1046/j.1365-313X.2002.01297.x> |
|  | Malus domestica | MdNPR1 | ANJ45434.1 | <https://doi.org/10.1007/s11295-016-1050-7> |
|  |  | MdNPR2 | KU166913 |  |
|  |  | MdNPR3 | ANJ45437.1 |  |
|  |  | MdNPR4 | ANJ45438.1 |  |
|  |  | MdNPR5 | ANJ45443.1 |  |
|  |  | MdNPR6 | ANJ45444.1 |  |
|  |  | MdNPR7 | ANJ45440.1 |  |
|  |  | MdNPR8 | ANJ45441.1 |  |
|  | Vitis vinifera | VvNPR1.1 | XP_002281475 | <http://doi.org/10.1186/1471-2229-9-54> |
|  |  | VvNPR1.2 | XP_003633057 |  |
|  | Populus trichocarpa | PtNPR1 | XP_002308281.1 | <https://doi.org/ 10.1111/jph.12002> |
|  |  | PtNPR2 | XP_002322351.2 |  |
|  |  | PtNPR3 | XP_002300863.2 |  |
|  |  | PtNPR4 | XP_002307566.2 |  |
|  |  | PtNPR5 | XP_002323261.1 |  |
|  |  | PtNPR6 | XP_002308905.1 |  |
|  | Carica papaya | CpNPR1 | AAS55117.1 | <http://doi.org/10.1007/s13258-011-0218-7> |
|  |  | CpNPR2 | XP_021890039.1 |  |
|  |  | CpNPR3 | XP_021898330.1 |  |
|  |  | CpNPR4 | XP_021908418.1 |  |
|  | Persea americana | PaNPR1 | AKH61407.1 | <https://doi.org/10.3389/fpls.2015.00300> |
|  |  | PaNPR2 | AKH61408.1 |  |
|  |  | PaNPR3 | AKH61409.1 |  |
|  |  | PaNPR4 | AKH61410.1 |  |
|  |  | PaNPR5 | AKH61411.1 |  |
|  | Cocos nucifera | CnNPR1 | AOW69311.1 | <http://doi.org/ 10.1007/s13258-017-0566-z> |
|  |  | CnNPR3 | AMD39572.1 |  |
|  | Glycine max | GmNPR1-1 | FJ418595 | https://doi.org/10.1186/1471-2229-9-105 |
|  |  | GmNPR1-2 | FJ418597 |  |
|  | Capsicum annuum | CaNPR1 | NP_001312028.1 | <https://doi.org/10.3724/SP.J.1142.2012.50494> |
|  | Gossypium hirsutum | GhNPR1 | EF988657 | https://doi.org/10.1042/BSR20070028 |
|  | Citrus sinensis | NPR1 | XP_006475479.1 | <http://ddoi.org/10.1007/s10658-010-9633-x> |
|  | Pyrus pyrifolia | PpNPR1-1 | ABK62792 | <http://doi.org/10.1080/00949651003724790> |
|  | Morus multicaulis | MuNPR1 | AGI03909.1 | <https://doi.org/10.1111/ppl.12889> |
|  |  | MuNPR4 | AGL61506.1 |  |

**Table S 2.**  List of primers used for qRT-PCR analysis and BiFC vector construct and their sequence

| **Gene name** | **Primer sequence** |
| --- | --- |
| ACTIN | F: TTGGTATGGGTCAGAAGG  R: CTGTGAGCAGAACTGGGTG |
| Pbrgene12425 | F: AGGA TACACAGGGC TTTGGATTCG  R: AACACAGTGT ACCCCCTCGG ATTC |
| Pbrgene8895 | F:GATACGTCCTTAGGCTATACT  R:GATCAACCAACAACAGCTCCA |
| Pbrgene8896 | F: CCCTGGAGCTAATAAACGTGG  R: CATGGAGATTAACGAGATGGATC |
| Pbrgene8341 | F: GTCCACGTGCGTTGATGATG  R: CTCCACAAAGTTTGAAAGACGG |
| Pbrgene6286 | F: GCGGAGACATCTGAGTTTGC  R: CCAGGACTTCCGAGCAATGA |
| Pbrgene34018 | F: TGTACCACCACTCTCACGACT  R: CCACTACCGATTAATTAATCTCCTTAGAC |
| Pbrgene2529 | F: GCGGAGACATCTGAGTTTGC  R: CCAGGACTTCCGAGCAATGA |
| Pbrgene40077 | F: GGGATTGGAAAGGGGAAGATCCC  R: GCATTGCACCTCCGCCGTTGATCTTG |
| Pbrgene12425YFP | F: CAGGCCTGGCGCGCCACTAGTATGGGTGACGATCACTTCGT  R: ACTATCGATGGATCCACTAGTTTTCTTGATGGTGATCGTAC |
| Pbrgene6286YFP | F: CAGGCCTGGCGCGCCACTAGTATGGCTCATTCAGCCGAACC  R: ACTATCGATGGATCCACTAGTCGGTTCCCTAACCTTCTGAT |
| Pbrgene8895YFP | F: CAGGCCTGGCGCGCCACTAGTATGGCTTATTCAGCTGAACC  R: ACTATCGATGGATCCACTAGTTTTCCTAACCTTCTGATTTG |

**Table S3. Analysis and distribution of conserved motifs in *PbrNPR1*-like proteins.**

| **Motif** | **P-Value** | **Site** | **Width** | **Best possible match** |
| --- | --- | --- | --- | --- |
| 1 | 2.0e-318 | 14 | 50 | LDSDDVELVKLLLTESGJTLDEANALHYAVAYCDPKVVKELLELGLADVN |
| 2 | 6.3e-289 | 14 | 50 | RNPRGYTVLHIAAMRKEPSIIVVLLDKGADASELTSDGRTAVDICRRLTR |
| 3 | 7.4e-237 | 14 | 41 | CVDDGCAHDACRPAIDFAVELLYASSVFQVPELVSLFQRQL |
| 4 | 2.2e-152 | 10 | 44 | GSSGNLTEVDLNETPFVQTKRLLSRLEALMKTVELGRRYFPHCS |
| 5 | 1.5e-140 | 14 | 23 | GRPVGVHRCILAARSLFFRELFK |
| 6 | 8.0e-178 | 14 | 41 | KPKYQMSELLPYGSVGYEAFLVFLSYLYSGKLKPVPKEVST |
| 7 | 2.1e-183 | 14 | 41 | HCDLHQLLDQCIERVAKSDLDPESJEKELPIEVVKKIKELR |
| 8 | 4.0e-162 | 10 | 50 | EREMRRNPMAGDASISSQIMPDDLHMRLLYLENRVALARLLFPAEAKLAM |
| 9 | 1.9e-126 | 14 | 36 | PDLFYLEPGTTEERRNKRRRYLELKEDVQKAFSKDK |
| 10 | 4.1e-053 | 14 | 15 | NFVEKALIEDVLPIL |

**FigS. 1:** Logo of ten conserved motifs of PbrNPR1-like proteins obtained through MEME online server


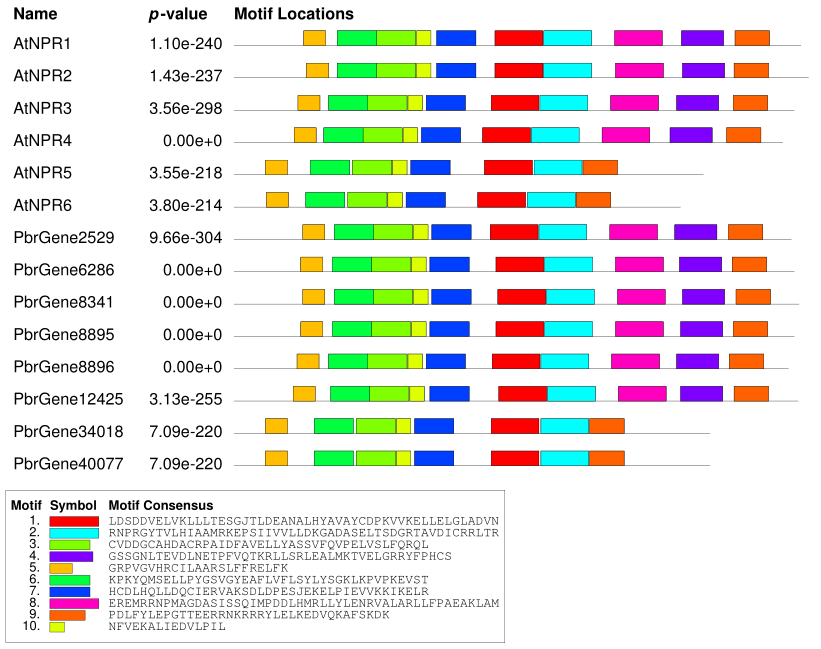


**Table S4:** Putative cis-elements identified in *PbrNPR1-like* genes using PlantCARE database

| **Element** | **Sequence** | **Function** |
| --- | --- | --- |
| ABRE | ACGTG | cis-acting element involved in the abscisic acid responsiveness |
| AE-box | AGAAACTT | part of a module for light response |
| ARE | AAACCA | cis-acting regulatory element essential for the anaerobic induction |
| ATCT-motif | AATCTAATCC | part of a conserved DNA module involved in light responsiveness |
| Box 4 | ATTAAT | part of a conserved DNA module involved in light responsiveness |
| CGTCA-motif | CGTCA | cis-acting regulatory element involved in the MeJA-responsiveness |
| G-Box | CACGTT | cis-acting regulatory element involved in light responsiveness |
| GARE-motif | TCTGTTG | gibberellin-responsive element |
| GATA-motif | GATAGGA | part of a light responsive element |
| GT1-motif | GGTTAA | light responsive element |
| I-box | gGATAAGGTG | part of a light responsive element |
| LTR | CCGAAA | cis-acting element involved in low-temperature responsiveness |
| MBS | aaaAaaC(G/C)GTTA | MYB binding site involved in drought-inducibility |
| MRE | AACCTAA | MYB binding site involved in light responsiveness |
| MYB | TAACCA | MYB binding site |
| MYC | CATTTG | MYBCbinding site |
| O2-site | GATGA(C/T)(A/G)TG(A/G) | cis-acting regulatory element involved in zein metabolism regulation |
| P-box | CCTTTTG | gibberellin-responsive element |
| STRE | AGGGG | Pressure responsivness |
| TATC-box | TATCCCA | cis-acting element involved in gibberellin-responsiveness |
| TC-rich repeats | GTTTTCTTAC | cis-acting element involved in defense and stress responsiveness |
| TCA-element | TCATCTTCAT | cis-acting element involved in salicylic acid responsiveness |
| TCCC-motif | TCTCCCT | part of a light responsive element |
| TCT-motif | TCTTAC | part of a light responsive element |
| TGA-element | AACGAC | auxin-responsive element |
| TGACG-motif | TGACG | cis-acting regulatory element involved in the MeJA-responsiveness |
| W box | TTGACC | fungal elicitor responsive element |
| WUN-motif | AAATTTCCT | wound-responsive element |
| as-1 | TGACG | Disease-associated protein binding sites |
| circadian | CAAAGATATC | cis-acting regulatory element involved in circadian control |
